# Communities in world input-output network: Robustness and rankings

**DOI:** 10.1371/journal.pone.0264623

**Published:** 2022-04-25

**Authors:** Alexei Kireyev, Andrey Leonidov, Stanislav Radionov, Ekaterina Vasilyeva

**Affiliations:** 1 International Monetary Fund, Washington, DC, United States of America; 2 Theoretical Physics Department, P.N. Lebedev Physical Institute, Moscow, Russia; Politecnico di Milano, ITALY

## Abstract

We introduce a method for assessing the robustness of community detection and apply it to a world input-output network (WION) to obtain economically plausible results. This method enabled us to rank communities in the WION in terms of their robustness and stability. The algorithmic assignment variability index proposed in this study is shown to have predictive power in terms of forthcoming community rearrangement. We also provide several new approaches for identifying key economic communities. These approaches are based on the application of several centrality measures to a synthetic network in which nodes represent WION communities. Using these methods, we show that in 2000–2014, United States and Japan-centered communities demonstrated decreasing trends, while the importance of the China-centered community predominantly increased. A notable feature of the Germany-centered community rank evolution is that its influence grew only as a result of the inclusion of the Netherlands and Belgium in 2013.

## Introduction

In recent years, the global economy has been increasingly analyzed as an economic network. This became possible after the publication of multi-country input-output tables, such as the world input-output database (WIOD) [1], which generalized Leontieff input-output tables [2] to a multi-country case and included data on bilateral cross-country trade in intermediate inputs and final goods. Multi-country input-output tables have been used to analyze trade in terms of the value added ([3–6]), global value chains (GVCs) ([7–11]), role of individual countries in GVCs ([12, 13]), and other topics.

WIOD data on trade in intermediate inputs can be presented in the form of an adjacency matrix as a world input-output network (WION). In such a network, the nodes correspond to sectors of different countries, the edges reflect the direction of trade, and their weights are proportional to trade values. The properties of WIONs have also been extensively studied. Some authors have focused on the GVC dimension using topological metrics [14, 15], competitive advantages of individual countries using graph theory [16], and structural changes using the centrality vector measure [17]. Other authors have focused on the search for key sectors in global production networks by using the betweenness centrality measure [18] and the weighted degree centrality measure [19]. Some used communicability distances to measure the interactions among countries [20].

The objective of this study is to develop a new method for the identification of robust economic communities. At the global level, such communities could be considered as new economic agents, along with countries and firms, on the grounds that they create value added as integrated units. Several papers have studied trade networks with the aim of detecting communities in value-added creation [14, 15, 21–23]. For example, in [14], the authors studied the community structure of GVC networks in 1995, 2003, and 2011 using the Newman and Girvan algorithm [24]. They showed that the community structure was effectively country-centered, and trade intensity was mainly determined by geographical proximity. The only evident globalization trend appears in Europe around the German economy. In [15], the authors introduced a new synthetic network in which nodes represented communities, and the trade flows among their members were aggregated into links. A similar idea was proposed in [21], where nodes represented countries and the links reflected cross-country flows. The substantive difference between such a synthetic network derived from WION and the world trade network (WTN), where nodes represent countries and edges are import/export flows, is the existence of self-loops, which are important for the detection of the central nodes.

Community partition algorithms are often based on the maximization of an objective function, such as modularity. However, in many real-world networks, there may exist many partitions with a relatively high objective function value (see [25] and references therein). As noted in [25], such partitions are meaningful and generally not inferior to those that maximize an objective function value. In addition, in the community detection process, certain combinations of nodes may remain together, even when they migrate among communities. Therefore, such node combinations can be considered as building blocks for community partition.

The relative importance of WION communities has also been assessed based on centrality measures, either for WION as a whole or its aggregated synthetic versions. For example, some authors studied centrality vectors for the GVC network ([14, 15, 17–19, 21, 26]), where nodes correspond to countries, and ranked their country-sector pairs. Other studies calculated node centralities in more aggregated networks, where nodes corresponded to countries, and edges represented exports, imports, or intra-country trade flows (self-loops). While both approaches are possible, they provide different perspectives on the network structure of trade. While the former helps identify dominant global players, the latter provides a more general view of the distribution of economic powers.

This paper contributes to the literature along several dimensions. This paper:

- Proposes several improvements to the community detection algorithm in application to WION, which allows for economically plausible results to be obtained. In particular, the study shows that countries play the role of basic building blocks in economic communities, similar to [25].
- Applies two measures of community detection robustness (with respect to the algorithm internal randomization and edges’ weights perturbation) and shows that the one corresponding to the algorithm internal randomization may have predictive power in terms of forthcoming community rearrangement. The robustness of community detection in a number of complex networks using different indicators was addressed in [27–32]. However, to the best of our knowledge, this is the first application of these two measures in the quantitative analysis of robustness of community detection in economic networks.
- Analyses centrality measures for aggregated synthetic networks, where nodes correspond to communities, rather than countries.

## Data and methods

This study is based on the 2016 release of the WIOD [1]. It includes data for 2000–2014 on 56 sectors in 43 advanced countries and emerging economies, and an estimate for the rest of the world.

**Fig 1:**
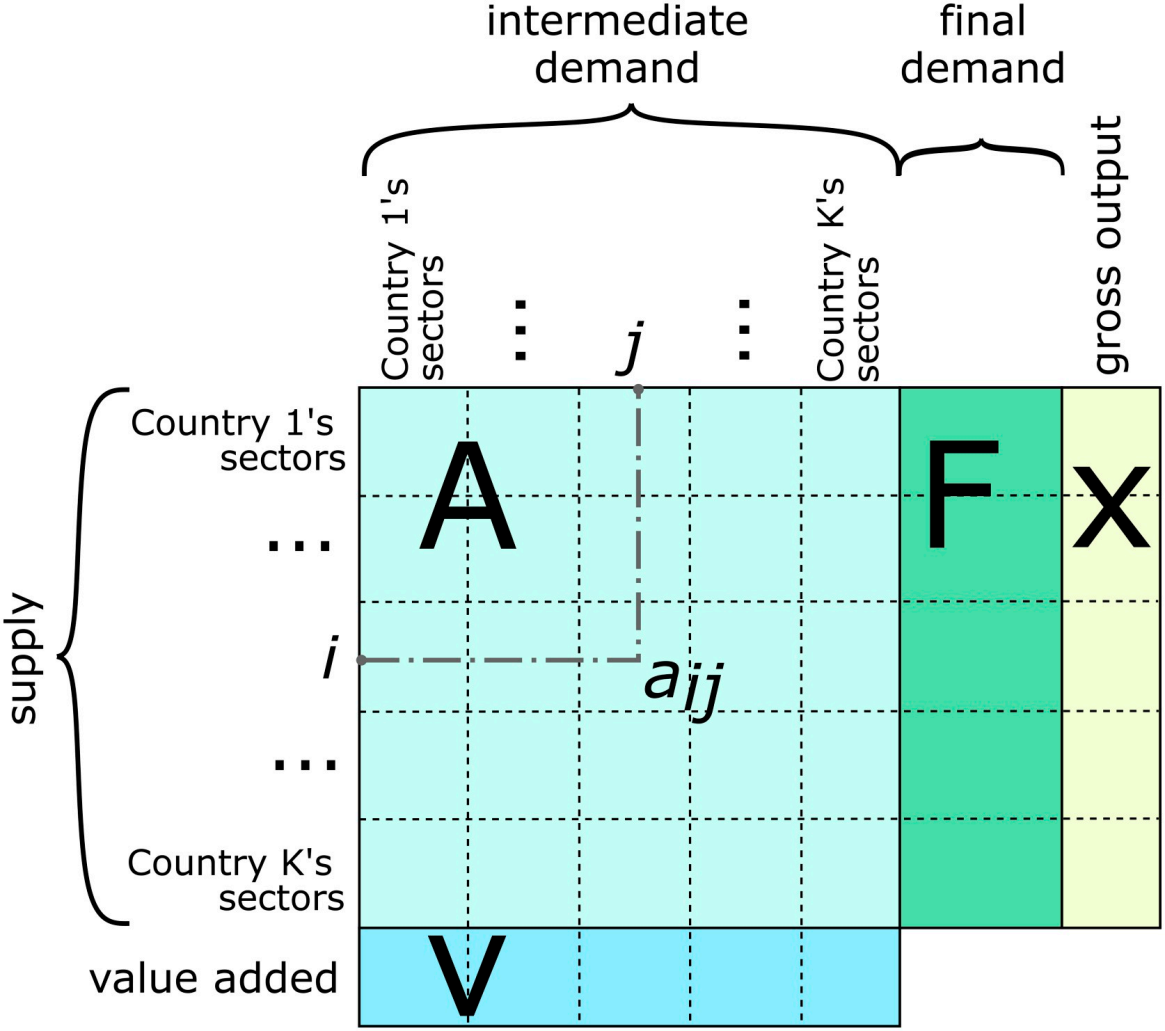
Structure of WIOD tables.

The input-output tables for each year have identical structures, as shown in Fig 1.

Structurally, the table includes a matrix of trade in intermediate inputs between country-sector pairs **A**, a matrix of final demand (use) by sector **F**, as well as a gross output vector **x** and a value-added vector **v**.

The matrix elements *a*_*ij*_ in **A** correspond to the flows of intermediate inputs produced by country *i* and are used in country sector *j*. Therefore, row *i* shows sector *i*’s sales of intermediate inputs, and column *j* in the matrix represents country-sector *j*’s intermediate demand for these inputs. The elements of matrix **F** correspond to the final demand for goods produced by a country sector and their use in different countries. Vector **x** is the gross output of a country sector, which, by construction, is equal to the sum of all elements of **A** and **F** in the corresponding row. The elements of vector **v** are the value added of country-sectors for columns of **A**.

From this input-output table, we construct the adjacency matrix *W* for the WION.

$$\begin{array}{c} W=(\begin{array}{cc} A & F\\ \text{0} & \text{0} \end{array}). \end{array}$$ (1)

**Fig 2:**
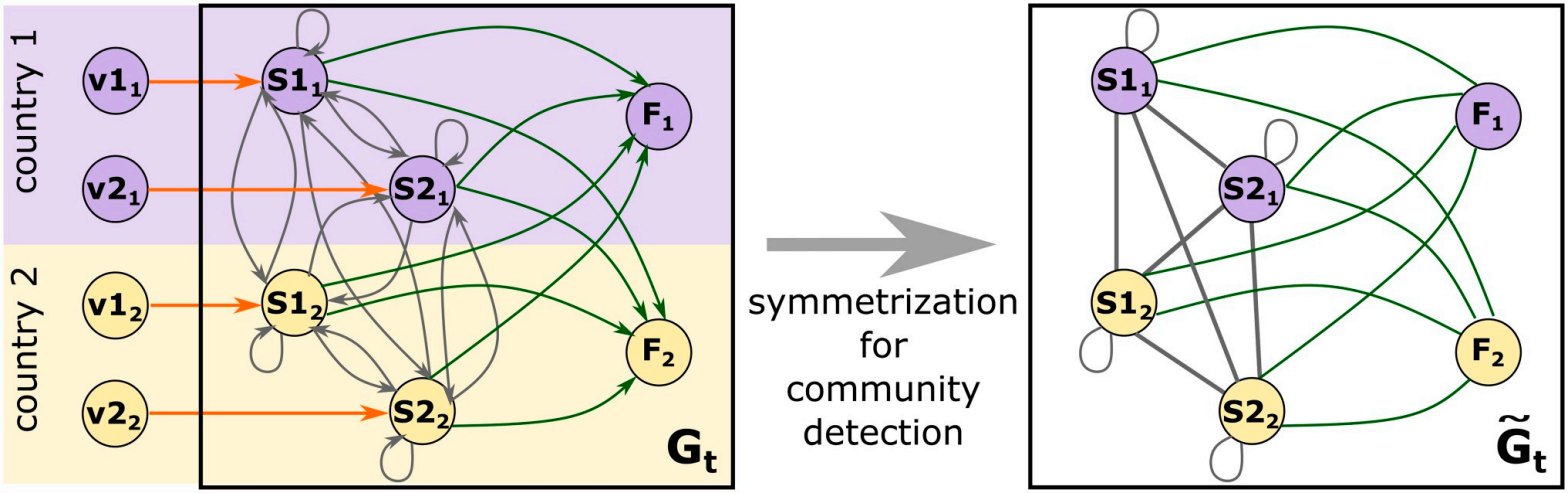
A two-sector two-country world economy network [14]. Node *Si*_*j*_ (*i*, *j* = 1, 2) is country *j* sector *i*; *F*_*j*_ (*j* = 1, 2) is country’s *j* final demand, and *vi*_*j*_ (*i*, *j* = 1, 2) is the value-added of country *j* sector *i*.

In contrast to previous studies [14], our *W* includes matrix *F* (final demand). Accurate community assignment is required, particularly for small country-sector nodes. Matrix **A** can be viewed as an adjacency matrix of the WION subgraph, where nodes represent country-sector pairs and edges, the flows of intermediate inputs. In turn, matrix *F* is a WION subgraph adjacency matrix with a bipartite structure. It links country-sector supply pairs to final demand nodes with one aggregate sector per country. The structure of this network is illustrated in the left panel of Fig 2 [14] for the case of a two-sector and two-country world economy.

In these networks, $G_{t}=\left(N_{t},E_{t},W_{t}\right),\;t=2000,\ldots ,2014$, $N_{t}$ and $E_{t}$ are the sets of nodes and edges, respectively, and **W**_*t*_ are the matrices of the edge weights. The nodes represent country-sector supply pairs or country demand pairs, the edges show the direction of product flows, and their weights $w_{ij}^{t}$ are the values of products, where node *i* is sold to node *j*. The final demand nodes have only incoming links. In the preprocessing stage, the sectors with zero inputs and outputs are excluded, as they create redundant isolated nodes in the community search algorithm (see Fig 8 in [14]).

As every edge in the WION carries information on two opposite flows (the product and financial ones) to quantify the intensity of the connection between two county-sector nodes, the network weight matrix **W** is symmetrized by summing up the weights in the opposite directions (right panel, Fig 2). To detect communities in this symmetrized network, we use the modularity-maximizing Louvain algorithm [33].

An important feature of the Louvain algorithm is the use of heuristics in modularity optimization. The result depends on the assumption of the initial community partition and the choice of the node sequence used in an algorithm call (for technical details, see [33]). The community partitions corresponding to the highest modularities can thus vary from call to call. To the best of our knowledge, such partition variability for real-world networks is quite common [25], but its effects on community detection in economic networks have not yet been studied. This fact should be considered when assessing the quality of community assignments.

To address the first issue, we identified the appropriate assumption of the initial community partition. For this, three common assumptions were considered:

- a trivial partition, where each community contains only one node;
- a country-based partition, where all sectors of a country belong to the same community;
- a previous year partition, where the current year partition is equivalent to the previous year partition (for each year except for 2000).

The assumption of the initial partition can lead to significantly different community assignments. In the first case, the resulting community structure varied significantly from year to year. In the second case, the resulting communities were slightly different from the first case, but with lower inter-year variability. Finally, in the third case, the community structure remained broadly unchanged from year to year.

To address the second issue of dependence on the node sequence used in an algorithm call, the following method was used. We ran a sequence of algorithm calls for each assumption in the initial configuration. The call with the highest modularity value would indicate the best assumption of the community partition for a given year. Specifically, we ran 90 algorithm calls for each year from to 2000–14 under different assumptions on the initial community partition. For 2000, we ran 45 calls under the assumption of a trivial initial partition and 45 calls under the assumption of a country-based partition. For 2001–14, we ran 30 calls under each of the three assumptions. From the ranges of the resulting modularity values (S1 Table), we chose the partition with the highest modularity value as the main partition. This indicates the best assumption on the initial partition for a given year. For example, for 2003, this was a trivial partition; for 2008, this was the country-based partition; and 2010, this was the previous year’s partition (see S2 Table for a complete list of selected initial partitions).

## Results and discussion

### Community stricture of the global economy

After selecting the initial partitions, we ran the Louvain algorithm and found 26 communities of nodes representing country-sector pairs in the WION. These communities have several important features.

First, most sectors of one country belong to the same community; therefore, they are not divided among different communities in sizeable proportions (in terms of numbers of sectors or shares of value added). This outcome is predictable, as the economic links between sectors in a specific country are usually sufficiently tight to pull them into the same community. Using the terminology of [25], in our network, countries may be described as the “building blocks”—the sets of nodes appearing together in different communities with high modularity. The only country that is close to being an exception is Luxembourg. In particular, in 2003, 42 out of 54 sectors belonged to one community, while the 12 remaining sectors were distributed among other communities. In 2008, 40 sectors belonged to one community and 14 to other ones; in 2011, there were 37 sectors in one community and 14 in others. However, in all these cases at least 85% of the value added was concentrated in a “leading” community.

Second, sometimes the countries are so strongly economically connected that the tightness of inter-country links becomes comparable to intra-country links. Therefore, several countries may constitute a single community. However, as noted in [14], most communities are single-country communities. By contrast, it is common for some sectors of one country to fall into a community formed predominantly by sectors of a different country. These are usually export- or import-dependent sectors.

Summarizing these two findings, the resulting typical community can be described as containing all or almost all sectors of one or several counties and sometimes a few sectors of other countries. For example, in 2000, one such community included all sectors of Ireland and all sectors of Great Britain except for “Water Transport” as well as “Mining and quarrying” of Norway and “Manufacture of other transport equipment” of Luxembourg.

Third, in all identified communities, the largest contributor to value added has remained unchanged, even if the membership of communities has changed. Therefore, a community is labeled after a country makes the highest contribution to its value added. Throughout the paper, we will use the three-letter ISO code of the corresponding country as the name of the community (see S3 Table). For instance, in the above-described example of a typical community, the label is GBR. In general, one can consider the evolution of a community with a fixed label but different memberships over the years. Discussing this dynamic, we will, for the most part, neglect the transitions of separate sectors from one community to another and analyze the situation when (almost) all sectors of a given country move from one community to another (more detailed data are available upon request).

Fourth, countries may change their community memberships. This happens when most or all of their sectors shift from one community to another. The structure of the BRA (the only case) did not change over 2000—14; the composition of 14 communities (USA, FRA, JPN, ITA, CAN, MEX, ESP, CHN, KOR, TWN, AUS, IND, IDN, ROW) varied only in several country-industry pairs from year to year. All these communities, except for ESP, are single-country communities, while the ESP consists of two countries, Spain and Portugal. The remaining 11 communities (DEU, NLD, HRV, POL, GBR, SWE, RUS, CHE, GRC, TUR, and ROU) were more dynamic.

**Fig 3:**
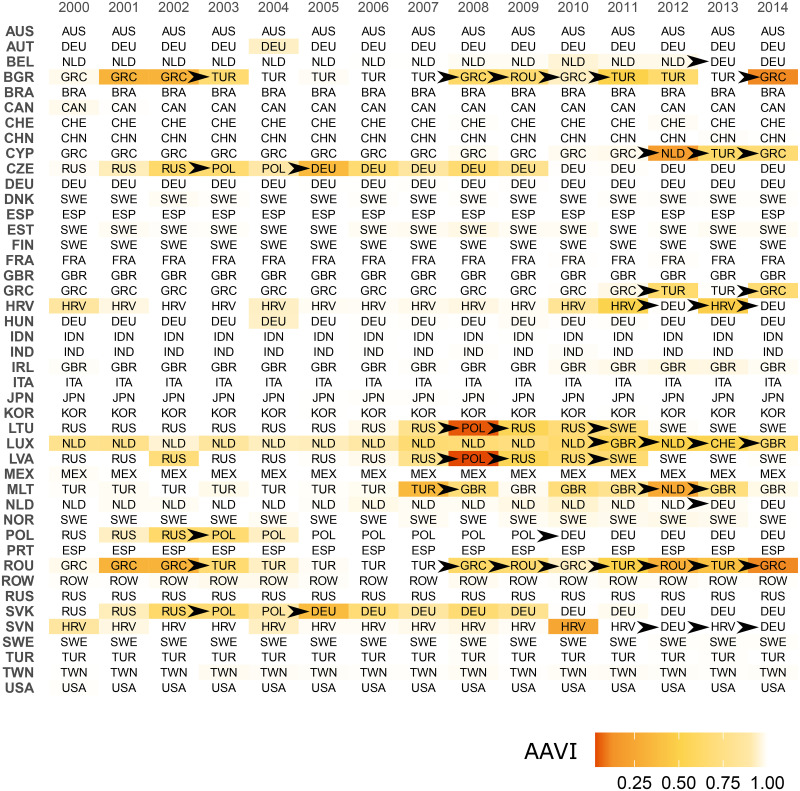
Assignment of countries to communities. The algorithmic assignment variability index (AAVI) is defined in Eq (3)). An AAVI value close to unity (light colors) indicates more reliable assignments. Black arrows highlight the cases in which the country changes its community.

Fig 3 shows the annual assignment of the majority of sectors of a given country to a particular community. A complete information on the annual country-sector assignments is available upon request. Arrows in some cells of the table in Fig 3 indicate transitions of a country from one community to another. The memberships of the identified communities for 2000, 2007, and 2014 is also provided in S4 Table.

A closer look at the temporal evolution of WION communities leads to several conclusions.

First, some communities can be considered transitional, as they ultimately join other communities. For example, the NLD community in 2000–10 included Belgium, the Netherlands, and Luxembourg. Later, in 2011, Luxembourg left the community. In 2013, this community disappeared, as Belgium and the Netherlands joined the DEU community. The POL community first appeared in 2003 when Poland, the Czech Republic, and Slovakia separated from the RUS community. The POL community disappeared in 2010 when all members joined the DEU community. In 2000–2010, the HRV community consisted of Croatia and Slovenia. In 2011, Slovenia joined the DEU community. Croatia followed in 2012. In 2013, a separate community consisting of Croatia alone emerged, but in 2014, Croatia rejoined the DEU community. All these transitions point to the long-term trend toward building a Germany-centered economic community ([14]).

Second, a gradual disintegration of the Russian economic community was observed. In 2000, the RUS community included Slovakia, Lithuania, Russia, the Czech Republic, Poland, and Latvia. In 2003, Slovakia, the Czech Republic, and Poland left the community. In 2008, Lithuania and Latvia joined the POL community. In 2009, both countries temporarily returned to the RUS community and joined the SWE community, consisting of Denmark, Sweden, Finland, Estonia, and Norway.

Third, the community assignments of some countries are highly unstable, particularly in smaller countries. For example, Turkey, Romania, Bulgaria, Cyprus, and Greece have often changed their communities between TUR, GRC, and ROU, with the latter two communities occasionally appearing and disappearing. This makes the TUR, GRC, and ROU communities dynamically unstable. In addition, Malta left the TUR community in 2008 but has been assigned to the GBR community since then.

### Algorithmic community assignment variability

The quantitative assessment of the inherent algorithmic robustness of community assignment is based on the following method: Inspired by [25], in addition to detecting the partition with the highest modularity, we analyzed the inner structure of partitions generated in a set of algorithm calls. Such partitions can be qualitatively viewed as a series of “local maxima” in the space of different network divisions. Therefore, these “local maxima” are also meaningful for illustrating the existence of competing assignments. (See the analysis of local partition stability in the S1 Appendix).

To measure the robustness of community assignment, one needs to introduce the distance between two partitions and a quantitative measure that would allow the assessment of the variability of partitions originating from different algorithm calls. Several approaches for defining such a distance have been proposed. These approaches can be roughly divided into three groups: those based on counting pairs, set matching, and variation of information (see [34]). In the problem under consideration, the structural properties of the community detection results allow us to use the simplest and, therefore, easily tractable measures. The reason is that communities are labeled after the country making the highest contribution to its value added. Therefore, the change in community structure can be evaluated based on the affiliation of each particular node to a particular label. A comparison of the results obtained using different definitions of distance is an interesting problem for further studies.

Each partition C can be presented as a vector of length *N* equal to the number of nodes in the graph (the sum of the number of country-sector pairs and number of country-consumer nodes), in which each element is the name of the community to which the node is assigned. The distance between two partitions C and $C^{\prime }$ can be quantified as the Hamming distance $D(C,C^{\prime })$ between the corresponding vectors and is equal to the number of nodes with different community names in C and $C^{\prime }$. The proximity $p(C,C^{\prime })$ between the two partitions can be measured as the fraction of the nodes with the same assignment:

$$\begin{array}{c} p\left(C,C^{\prime }\right)=1-\frac{D(C,C^{\prime })}{N}. \end{array}$$

Table 1 illustrates this calculation for the case of a two-country and three-sector world economy.

**Table 1:** Example of the calculation of the Hamming distance and proximity between to different partitions for the two-country the-sector world economy.

Country	Sector	Node id	Assignment		Contribution to distance
			C	$C^{\left(i\right)}$	
1	1	1	A	A	0
	2	2	A	A	0
	3	3	B	A	1
	final user	4	A	A	0
2	1	5	B	C	1
	2	6	C	C	0
	3	7	C	C	0
	final user	8	C	C	0
**Hamming distance** $D(C,C^{\left(i\right)})$					2
**Proximity** $p(C,C^{\left(i\right)})$					0.75

A, B, C are community names.

An algorithmic partition variability index (APVI), a quantitative measure of the algorithmic assignment variability in terms of the diversity of the competing high-modularity partitions, can be calculated for each graph *G*_*t*_, *t* = 2000, …, 2014. To this end, we denote the set $\hat{S}$ of 90 partitions generated in a given set of algorithm calls by $\hat{C}=\left\{{\hat{C}}^{\left(1\right)},\ldots ,{\hat{C}}^{\left(\hat{S}\right)}\right\}$. (To simplify notation, the time index was dropped). Assuming that C is the partition from $\hat{C}$ characterized by the highest modularity value, the APVI can be defined as follows:

$$\begin{array}{c} APVI=\frac{\sum_{i=1,\ldots ,\hat{S},{\hat{C}}^{\left(i\right)}\neq C}p\left(C,{\hat{C}}^{\left(i\right)}\right)}{\hat{S}-1}. \end{array}$$ (2)

APVI (2) characterizes the inherent algorithmic partition variability. The closer the APVI is to 1, the more uniform the outcome of different algorithm calls are and, therefore, the more robust the dominant partition.

The arguments leading to the definition of APVI can be used for a detailed analysis of dynamic patterns. APVI can be decomposed into a set of similar indices for individual countries. For this purpose, the same Hamming distance for the assignment vectors corresponding to sectors and consumer nodes of different countries can be calculated. For instance, in the economy in Table 1, the distances between the assignment vectors of both countries are equal to 1, and the proximities are equal to 0.75.

Furthermore, let $\hat{C}\left(k\right)=\left\{{\hat{C}}^{\left(1\right)}\left(k\right),\ldots ,{\hat{C}}^{\left(\hat{S}\right)}\left(k\right)\right\}$ be the set of country *k*’s sector community assignments, and C(k) be country *k*’s sector assignments in the partition with the highest modularity. Then, a country *k*’s algorithmic assignment variability index (AAVI), which has been used in color labeling in Fig 3, can be defined as follows:

$$\begin{array}{c} AAVI\left(k\right)=\frac{\sum_{i=1,\ldots ,\hat{S},{\hat{C}}^{\left(i\right)}\neq C}p\left(C\left(k\right),{\hat{C}}^{\left(i\right)}\left(k\right)\right)}{\hat{S}-1}. \end{array}$$ (3)

**Fig 4:**
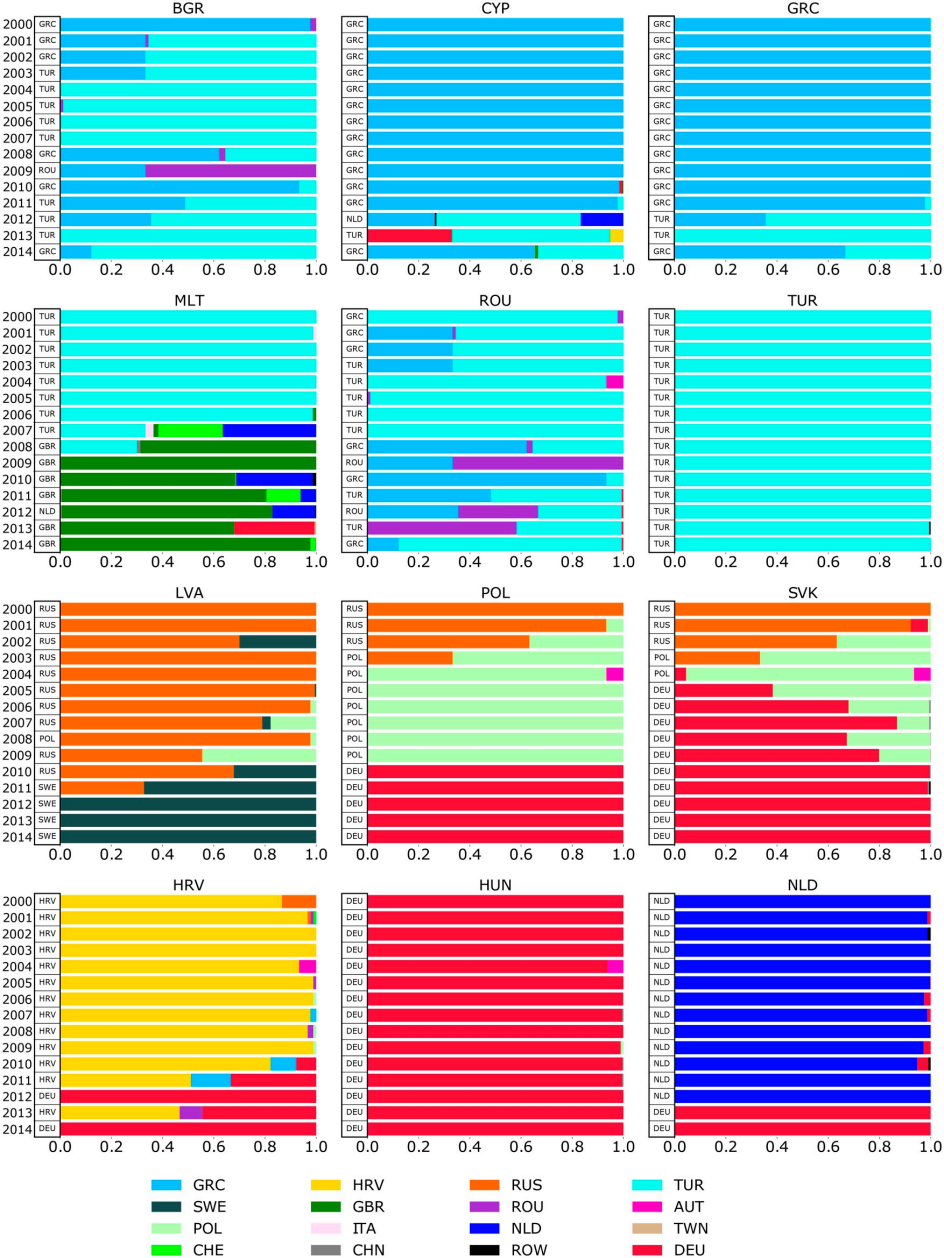
Fractions of algorithm calls due to the assignment of particular countries (an assignment of the majority of a given country sectors). The tables near the y-axis contain information about the assigned community in the partition with the highest modularity.

Fig 4 shows the relative weights of communities, to which several countries were assigned in different high-modularity partitions. For a given country and a given year, it shows the community to which it was finally assigned (shown in Fig 3) and the fraction of communities it was assigned to in different algorithm calls.

The first six plots in Fig 4 illustrate the complex dynamics of the TUR, GRC, and ROU community assignments. While Turkey’s assignment has been very stable, the reliability of other countries’ assignments seems questionable. For example, the share of algorithm calls assigning Bulgaria to the TUR community in 2002 and 2003 was the same. However, in 2002, in the main partition, Bulgaria was assigned to the GRC community, and in 2003, to the TUR community. The next four plots in Fig 4 illustrate the different cases of the transition dynamics. Hungary illustrates the case of simple dynamics, as almost all algorithm calls assign it to the DEU community. The last plot illustrates the simple transition dynamics, as almost all algorithm calls for 2000–2012 assigned the Netherlands to the NLD community, and all algorithm calls for 2013–2014 assigned it to the DEU community.

The dynamics of AAVI at the country level provide additional insights into the evolution of communities. AAVI is often low when a country changes its community. This is quite natural because the direction of trade of a country most likely changes slowly. During the transition period, it may not be clear to what community a country belongs, as illustrated by the cases of Poland and Slovakia in 2003.

In addition, a decrease in AAVI can be an indicator of the upcoming transition of a country to a different community. Empirically, AAVI often starts to decrease several years before a country changes its community. This can be seen in Poland. In 2000–2002, this country was assigned to the RUS community, but the share of algorithm calls pointing to this assignment has been decreasing. In 2003, Poland was assigned to the POL community, but the share of algorithm calls assigned to the RUS community was also high. Similar dynamics were observed for Slovakia. An exception can be illustrated by the case of the Netherlands’s transition from the NLD to the DEU community in 2013.

Hence, AAVI can be seen as a measure of assignment robustness, with a lower value indicating a lower reliability of country attribution to a particular community.

### Communities ranking

**Fig 5:**
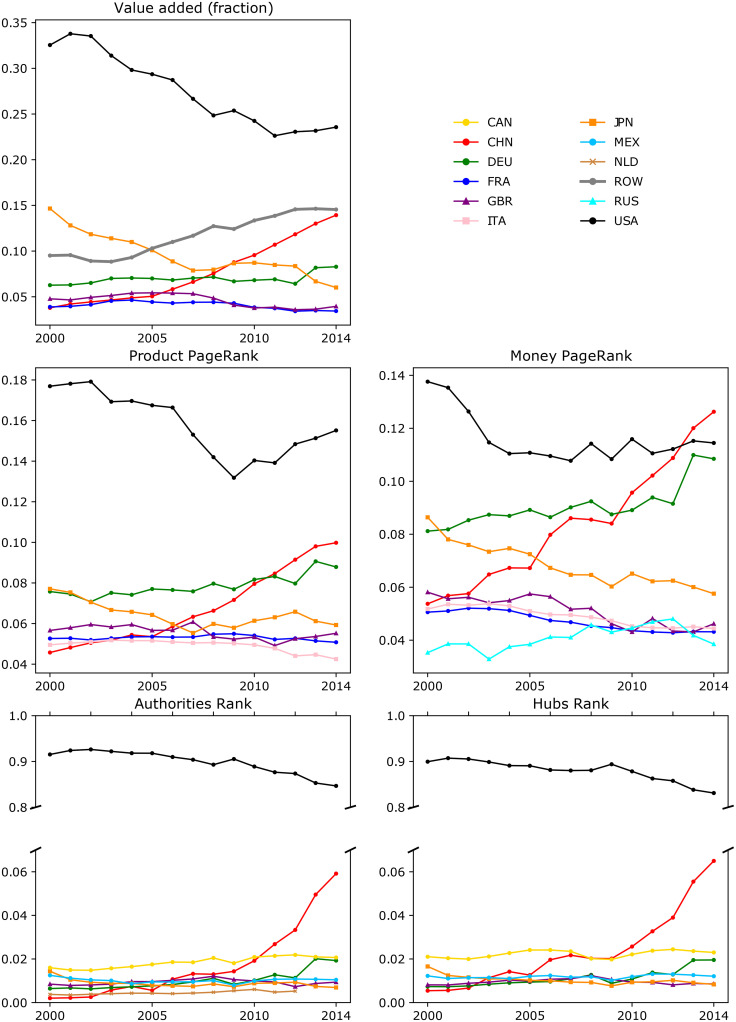
Ranking evolution of the top seven communities.

Ranking of communities may help establish their relative importance for the global economy. The value added created by each community is a natural metric for such a ranking. As the WIOD contains the value added for every country-sector pair, the total value added by the community is given by their aggregation. The first panel in Fig 5 shows the evolution of the value added for the top seven communities. Of these, only the DEU and GBR communities consist of more than one country. The shares of the global value added of the USA and JPN communities have been decreasing, while the shares of the ROW and CHN communities have been increasing. Significant growth in the DEU value-added share was observed only in 2013, after the Netherlands and Belgium joined it.

**Fig 6:**
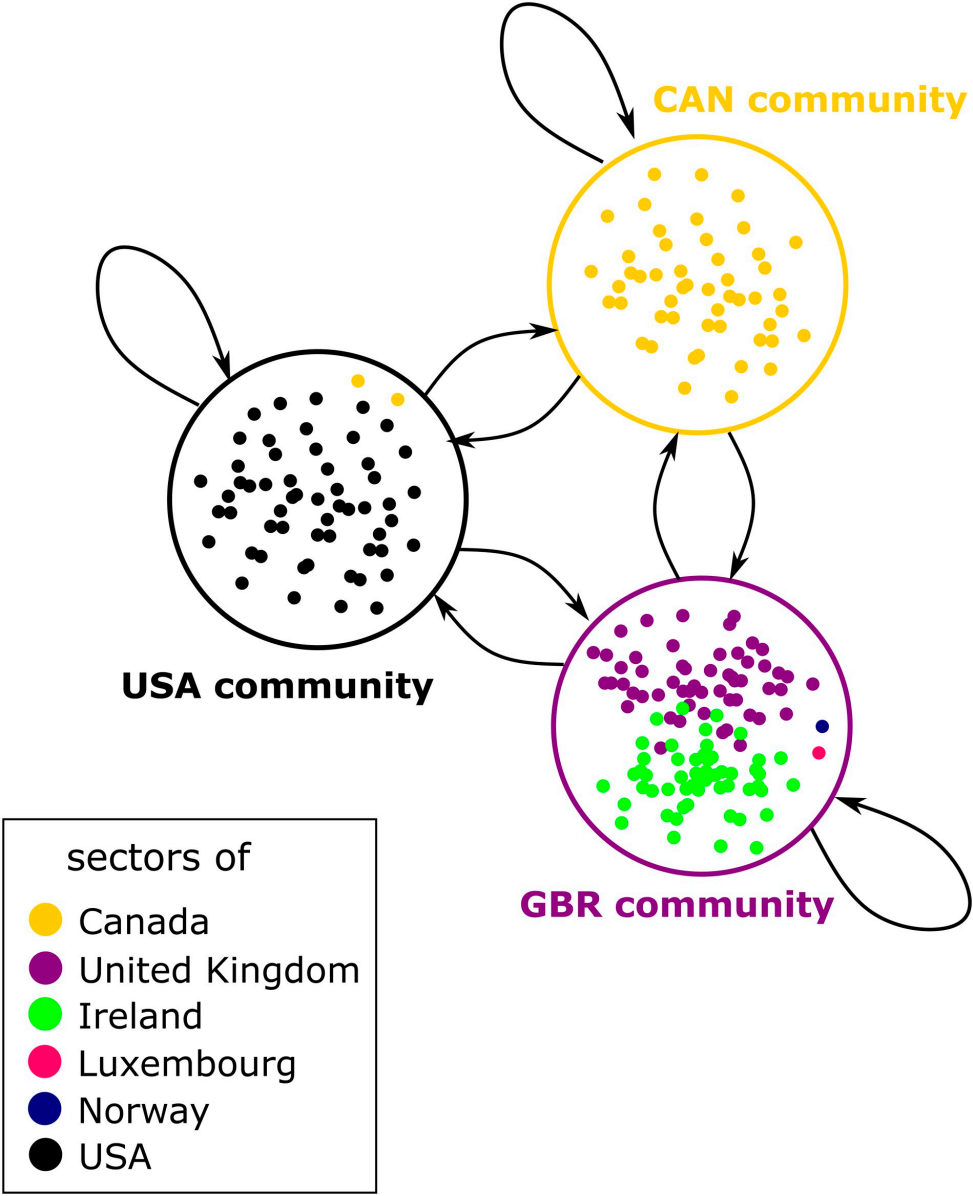
The fragment of world input output community network.

In addition, other community rankings have been proposed. For example, the identified communities, rather than countries, may be considered as the nodes of a network, which we call the world input-output community network (WIOCN). Thus, we consider WIOCNs $P_{t}=\left(N_{t}^{c},E_{t}^{c},W_{t}^{c}\right),\;t=2000,\ldots ,2014,$ where $N_{t}^{c}$ is the set of nodes representing communities, $E_{t}^{c}$ is the set of edges between at least one pair of nodes related to different corresponding communities in *G*_*t*_ and elements $w_{ij,t}^{c}$ of the weight matrix $W_{t}^{c}$ aggregate flows between communities and self-loops. A fragment of this network is shown in Fig 6.

The rankings can be based on various centralities, such as PageRank, and hubs and authorities. However, we decided to drop the ROW community because it caused significant distortions in the calculations. As seen from the plot in the first panel in Fig 5, the ROW community has a significant value added share and, therefore, seems influential. However, the ROW nodes aggregate information about many other country-sector pairs that are not present in the WIOD. Therefore, instead of the influential ROW node, the correct graph should contain many other “small” nodes, which is essential for the calculation of centralities such as PageRank or hubs and authorities. The main principle of these algorithms is that the more influential the node’s neighbors, the more influential the node. Therefore, cases where small and often disconnected nodes are collected in one large node and cases where they remain disconnected are fundamentally different. Therefore, to avoid distortions, it makes sense to disregard these “small” nodes rather than introducing a large synthetic ROW node.

The PageRank algorithm was proposed in [35] to rank Internet pages and is naturally defined for directed graphs. The main idea of the algorithm is described by the iterative procedure at each step, in which pages give parts of their rank to the pages that they cite. The WIONs *G*_*t*_ and, consequently WIOCNs *P*_*t*_, are also directed graphs, and the PageRank algorithm has been previously used to rank WION nodes ([14, 21, 26]). However, the notion of the direction of “rank spreading” is not obvious. Each product flow is matched with a money flow in the opposite direction. As shown in [26], the vector of PageRank centrality, calculated for the direction of money flows, determines the equilibrium in static and dynamic multi-sector models ([36–40]). However, at the aggregate level, where nodes are communities, this interpretation is not correct, and the PageRank scores calculated for product and money flows should be treated as different node ranking methods.

To overcome this deficiency, we define the product PageRank and money PageRank algorithms. While the former would calculate centralities with respect to product flows, the latter would do so for money flows.

Let $N_{t}^{c}=\left|N_{t}^{c}\right|$ and $x_{i,t}^{p},\;x_{i,t}^{m},\;i=1,\ldots ,N_{t}^{c}$ be the product and money PageRank vectors defined as follows:

$$x_{i,t}^{p}=d\sum_{j=1}^{N_{t}^{c}}\frac{w_{ji,t}^{c}}{\sum_{k=1}^{N_{t}^{c}}w_{jk,t}^{c}}x_{j,t}^{p}+\beta ,\;i=1,\ldots ,N_{t}^{c},$$ (4)

$$x_{i,t}^{m}=d\sum_{j=1}^{N_{t}^{c}}\frac{w_{ij,t}^{c}}{\sum_{k=1}^{N_{t}^{c}}w_{kj,t}^{c}}x_{j,t}^{m}+\beta ,\;i=1,\ldots ,N_{t}^{c},$$ (5)

where *d* and *β* are parameters. The typical choice of *d* is 0.85, and the choice of *β* depends on normalization. In the results below, we normalize the values such that the vector elements sum to one.

The calculations of the PageRank-type centralities are presented in the second row of the plots in Fig 5. The left plot shows the dynamics of product PageRank. These dynamics can be compared with the one presented in [21], which analyzed the product page ranks for countries. The top six countries are broadly the same, but their dynamics and rankings differ significantly. In our results, the USA remains by far the most important node for the whole period, whereas in [21], China overtakes it in 2008 and 2009. In addition, for the DEU community, the product PageRank has been growing, while in [21], it has been declining. The reasons for the differences are that our WIOCN treats communities as nodes, includes final demand flows, and excludes the ROW from the calculations.

The right plot in the second row of Fig 5 depicts the evolution of the money PageRank centrality for the top seven communities. Their dynamics and rankings are very different from those of product PageRank. For example, in 2013 and 2014, the CHN community became the most important. Moreover, the inclusion of the Netherlands and Belgium in the DEU community in 2013 strongly affected its relative money PageRank. In 2013 and 2014, the CHN, USA, and DEU communities were ranked almost equally high. This differs from the ranking by value added and product PageRank, where the USA community is substantially more important than the CHN and DEU communities.

The difference between product PageRank and money PageRank is in the direction of rank spreading. In the case of product PageRank, the rank spreads from the producer to the final user, that is, if a highly ranked community sells a significant share of its value added to some other community, the latter also has a relatively high rank. In the case of money PageRank, the rank spreads from the final user to the producer. Namely, if some highly ranked community pays significant amounts for imports from another community, then the latter gets a high rank as well. Therefore, the product PageRank can be considered as reflecting the final user ranking and the money PageRank with producer ranking.

This role separation between producers and final users is explicitly reflected in two special centrality measures, the hubs & authorities centralities ([41]). In this algorithm, each node plays the dual role of a producer and final user, and key producers are connected with key final users, and vice versa. In the resulting ranking, key producers are called hubs, and key final users are called authorities.

The iterative procedure used to calculate these rankings is as follows: Let $a_{i,t}^{\left(k\right)}$ be node *i*’s authority rank at the *k*th stage of the algorithm, $h_{i,t}^{\left(k\right)}$ be its hub rank, and $a_{i,t}^{\left(0\right)}=h_{i,t}^{\left(0\right)}=1$. Then, the step of the *k*th algorithm is defined as follows:

$$h_{i,t}^{\left(k\right)}=\sum_{j=1}^{N_{t}^{c}}w_{ij}^{c}a_{j,t}^{\left(k-1\right)},$$ (6)

$$a_{i,t}^{\left(k\right)}=\sum_{j=1}^{N_{t}^{c}}w_{ji}^{c}h_{j,t}^{\left(k\right)}.$$ (7)

In addition, after each algorithm’s step, the ranks are normalized so that both rank vectors sum up to unity.

The resulting stationary distribution of the hubs & authorities rankings is shown in the last row of the plots in Fig 5. These rankings clearly differ from those described above. First, the USA community rank was much higher than the ranks of the other communities. In addition, US trading partners, such as the CAN and MEX communities, are now included in the top seven communities. For comparison, a recent study [42] also ranked countries in the WTN using the hubs & authorities algorithm. In 1992—2012, four countries (China, Germany, the USA, and Japan) were ranked close to the top in the hub ranking. Meanwhile, in the authority ranking, the USA ranked much higher than the rest of the communities, similar to our results. In the case of hub ranking, the reason for such differences is the existence of self-loops in the WIOCN. In particular, due to the heavy self-looped USA community, the high authority rank spreads to its hub rank, and then to the ranks of its geographical neighbors.

## Conclusion

This study investigated community detection in WION. We showed that the results of the Louvain community detection algorithm in WION strongly depend on the initial community partition and its internal randomization. Moreover, the community structure that maximizes modularity may not be the only reasonable community structure. To the best of our knowledge, this is the first time this fact was considered in detail in a recent study [25] and was neglected in the economic literature. Inspired by the results of [25], we propose several improvements of the community detection algorithm in application to WION and introduce APVI, which measures the difference between the community assignment with the highest modularity and other community assignments that provide the local maxima of the modularity function. We proceed by defining such an index on the country level, referred to as AAVI. This index has a very natural interpretation: a high valus means that the assignment of a given country to a given community is highly reliable and vice versa. A notable result of our study is that the AAVI value often decreases several years before there is a change in the country’s community, thus making it a leading indicator of this event.

We also provide several new approaches for identifying key economic players. These approaches are based on the application of several centrality measures to a synthetic network in which nodes represent identified WION communities with the highest modularity. Along with the share of the world value added, we calculated two variants of the PageRank and Hubs and Authorities ranks for this network. By analyzing the evolution of these rankings, we identified a series of notable trends in global economic force distribution.

## Supporting information

S1 Table Range of modularity values in the results of different algorithm calls. (PDF)

S2 Table Types of initial distributions used in the calls resulting in the main partitions. (PDF)

S3 Table ISO country codes. (PDF)

S4 Table Communities membership in 2000, 2007 and 2014. (PDF)

S1 Appendix Partial stability. (PDF)
