# Supplementary material for: Communities in world input-output network: Robustness and rankings
Source: PLoS One. 2022 Apr 25;17(4):e0264623. doi: 10.1371/journal.pone.0264623 (PMC9037945; doi:10.1371/journal.pone.0264623)
Supplement: S1 Table — (PDF) [file pone.0264623.s001.pdf]

S1 Table. Ranges of modularity values in results of different algorithm calls

	max	min
2000	0.7686	0.7669
2001	0.7693	0.7691
2002	0.7727	0.7727
2003	0.7816	0.7815
2004	0.7829	0.7828
2005	0.7823	0.7820
2006	0.7804	0.7800
2007	0.7841	0.7836
2008	0.7837	0.7832
2009	0.7971	0.7964
2010	0.7893	0.7886
2011	0.7841	0.7835
2012	0.7791	0.7784
2013	0.7754	0.7747
2014	0.7725	0.7713
