# Supplementary material for: Communities in world input-output network: Robustness and rankings
Source: PLoS One. 2022 Apr 25;17(4):e0264623. doi: 10.1371/journal.pone.0264623 (PMC9037945; doi:10.1371/journal.pone.0264623)
Supplement: S2 Table — (PDF) [file pone.0264623.s002.pdf]

S2 Table. Types of the initial distributions used in the calls resulting in the main partitions.

2000	2001	2002	2003	2004	2005	2006	2007
CNTR	PREV	PREV	TRIV	PREV	CNTR	PREV	PREV

2008	2009	2010	2011	2012	2013	2014	
CNTR	CNTR	PREV	CNTR	CNTR	CNTR	CNTR	

"TRIV" means the trivial initial partition, "CNTR" – the initial partition by countries, "PREV" – the previous year main partition.
