# Supplementary material for: Communities in world input-output network: Robustness and rankings
Source: PLoS One. 2022 Apr 25;17(4):e0264623. doi: 10.1371/journal.pone.0264623 (PMC9037945; doi:10.1371/journal.pone.0264623)
Supplement: S3 Table — (PDF) [file pone.0264623.s003.pdf]

S3 Table. ISO country codes

code	country	code	country
AUS	Australia	IRL	Ireland
AUT	Austria	ITA	Italy
BEL	Belgium	JPN	Japan
BGR	Bulgaria	KOR	South Korea
BRA	Brazil	LTU	Lithuania
CAN	Canada	LUX	Luxembourg
CHE	Switzerland	LVA	Latvia
CHN	China	MEX	Mexico
CYP	Cyprus	MLT	Malta
CZE	Czech Republic	NLD	Netherlands
DEU	Germany	NOR	Norway
DNK	Denmark	POL	Poland
ESP	Spain	PRT	Portugal
EST	Estonia	ROU	Romania
FIN	Finland	RUS	Russia
FRA	France	SVK	Slovakia
GBR	United Kingdom	SVN	Slovenia
GRC	Greece	SWE	Sweden
HRV	Croatia	TUR	Turkey
HUN	Hungary	TWN	Taiwan
IDN	Indonesia	USA	USA
IND	India	ROW	Rest of the world
