# Supplementary material for: Communities in world input-output network: Robustness and rankings
Source: PLoS One. 2022 Apr 25;17(4):e0264623. doi: 10.1371/journal.pone.0264623 (PMC9037945; doi:10.1371/journal.pone.0264623)
Supplement: S4 Table — (PDF) [file pone.0264623.s004.pdf]

S4 Table. Communities membership in 2000, 2007 and 2014.

	2000	2007	2014
AUS	Australia	Australia	Australia
DEU	Austria, Germany, Hungary	Czech Republic, Austria, Germany, Hungary, Slovakia	Czech Republic, Austria, Germany, Hungary, Slovakia, Belgium, Netherlands, Poland, Slovenia
NLD	Belgium, Luxembourg, Netherlands	Belgium, Luxembourg, Netherlands	
GRC	Bulgaria, Cyprus, Greece, Romania	Cyprus, Greece	Cyprus, Greece, Bulgaria, Romania
BRA	Brazil	Brazil	Brazil
CAN	Canada	Canada	Canada
CHE	Switzerland	Switzerland	Switzerland
CHN	China	China	China
RUS	Czech Republic, Lithuania, Latvia, Poland, Russia, Slovakia	Lithuania, Latvia, Russia	Russia
POL		Poland	
SWE	Denmark, Estonia, Finland, Norway, Sweden	Denmark, Estonia, Finland, Norway, Sweden	Denmark, Estonia, Finland, Norway, Sweden, Lithuania, Latvia
ESP	Spain, Portugal	Spain, Portugal	Spain, Portugal
FRA	France	France	France
GBR	United Kingdom, Ireland	United Kingdom, Ireland	United Kingdom, Ireland, Luxembourg, Malta
HRV	Croatia, Slovenia	Croatia, Slovenia	Croatia,
IDN	Indonesia	Indonesia	Indonesia
IND	India	India	India
ITA	Italy	Italy	Italy
JPN	Japan	Japan	Japan
KOR	South Korea	South Korea	South Korea
MEX	Mexico	Mexico	Mexico
TUR	Malta, Turkey	Bulgaria, Malta, Turkey, Romania	Turkey
TWN	Taiwan	Taiwan	Taiwan
USA	USA	USA	USA
ROU	formed only in 2009, when consisted of Bulgaria and Romania		
ROW	Rest of the world	Rest of the world	Rest of the world
