# Supplementary material for: Communities in world input-output network: Robustness and rankings
Source: PLoS One. 2022 Apr 25;17(4):e0264623. doi: 10.1371/journal.pone.0264623 (PMC9037945; doi:10.1371/journal.pone.0264623)
Supplement: S1 Appendix — (PDF) [file pone.0264623.s005.pdf]

S5 Appendix. Partition stability

Here we show that the results of a given algorithm call are stand for some "local maximum" and therefore represent existing tight links. To do this we analyse the stability of a given partition to small perturbations of the edges weights. We call this kind of partition variability the local stability and propose a formal measure to describe this phenomena called a local partition stability index.

Let us denote a particular partition of the network by $\mathcal{C} = \{C_1, \ldots, C_m\}$, where m is the number of communities, $C_k,\ k = 1, \ldots, m$, is the set of nodes in k-th community.

The exact approach used to characterize the local stability of a particular community structure $\mathcal{C}$ is the following. Let $\mathbf{W} = \{w_{ij}\}$ be a network weight matrix and $\widetilde{\mathbf{W}} = \{\tilde{w}_{ij}\}$ – its symmetric version (obtained by the summation of the opposite direction weights). Let also ε be some small value representing the relative perturbation intensity. Then the element $\tilde{w}_{ij}(\varepsilon)$ of the matrix $\widetilde{\mathbf{W}}(\varepsilon)$ is defined as follows:

$$\tilde{w}_{ij}(\varepsilon) = \tilde{w}_{ij} + \xi_{ij},\ \xi_{ij} \sim \mathfrak{N}(0, \varepsilon\tilde{w}_{ij}), \tag{1}$$

where $\mathfrak{N}(0, \varepsilon\tilde{w}_{ij})$ means normal distribution with standard deviation equal to $\varepsilon\tilde{w}_{ij}$.

Having some partition $\mathcal{C}$ and a sample of random weight-matrices $\widetilde{\mathbf{W}}^{(1)}(\varepsilon), \ldots, \widetilde{\mathbf{W}}^{(S)}(\varepsilon)$, where S is the sample size, one may obtain a series of partitions $\mathcal{C}^{(1)}, \ldots, \mathcal{C}^{(S)}$ where each partition is a partition of a graph with respective random matrix and prior nodes distribution equal to $\mathcal{C}$. We assume that $\mathcal{C}$ is locally stable if partitions $\mathcal{C}^{(1)}, \ldots, \mathcal{C}^{(S)}$ corresponding to relatively small numbers of ε are close to it. The qualitative term "close" can be defined formally using the concept of Hamming distance.

Using the proximity definition one may introduce the local partition stability index LPSI($\mathcal{C}|\varepsilon$) as the mean value of proximity between $\mathcal{C}$ and each of $\mathcal{C}^{(i)},\ i = 1, \ldots, S$:

$$LPSI(\mathcal{C}|\varepsilon) = \frac{\sum_{i=1}^{S} p(\mathcal{C}, \mathcal{C}^{(i)})}{S}. \tag{2}$$

Fig S5.1 shows the values of $LPSI(\mathcal{C}|0.05)$ and $APVI$. In addition, the diagram shows the sample quantiles (the areas between 0 and 25%, 25% and 75%, 75% and 100% quantiles) of $p(\mathcal{C}, \hat{\mathcal{C}}^{(i)}),\ i = 1, \ldots, \hat{S}$, distribution (the global proximity distribution). The same ranges for $p(\mathcal{C}, \mathcal{C}^{(i)}),\ i = 1, \ldots, S$, are not shown, as the intervals between minimal and maximal values for this sample are typically smaller then 1%. From this figure it is clear that local variability is much smaller than the global one. In Table S5.1 we also provide the values for the sample mean and minimal proximities for $\varepsilon = 1\%,\ 5\%$ and 10%. From here it is clear that partition $\mathcal{C}$ can be considered as locally stable, which justifies the presented analysis of the dynamics based on comparison of different years main partitions.

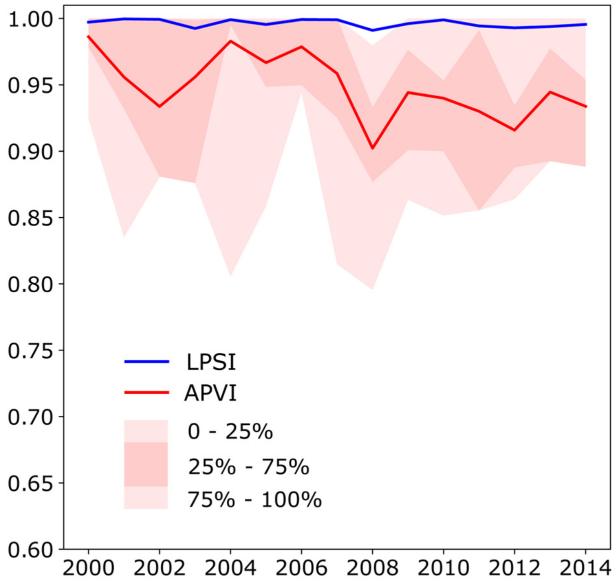

Fig S5. 1. The values of local partition stability index for $\varepsilon = 0.05$ $(LPSI(\mathcal{C}|\varepsilon))$ and algorithmic partition variability index $(APVI)$. The areas demonstrate the ranges between corresponding quantiles of the global proximity distribution.

Table S5. 1. Sample mean and minimal proximity values for the cases of $\varepsilon = 0.01,\ 0.05$ and 0.1.

	mean			min		
ε	**0.01**	**0.05**	**0.1**	**0.01**	**0.05**	**0.1**
2000	0.9975	0.9972	0.9969	0.9975	0.9962	0.9954
2001	1.0000	0.9997	0.9993	1.0000	0.9987	0.9983
2002	0.9997	0.9994	0.9989	0.9987	0.9983	0.9975
2003	0.9927	0.9925	0.9922	0.9924	0.9916	0.9903
2004	0.9999	0.9991	0.9987	0.9996	0.9979	0.9970
2005	0.9958	0.9955	0.9950	0.9949	0.9945	0.9933
2006	0.9997	0.9992	0.9988	0.9992	0.9983	0.9975
2007	0.9997	0.9991	0.9984	0.9987	0.9975	0.9970
2008	0.9916	0.9910	0.9905	0.9911	0.9899	0.9882
2009	0.9962	0.9961	0.9949	0.9962	0.9954	0.9494
2010	0.9995	0.9990	0.9944	0.9992	0.9975	0.9498
2011	0.9945	0.9944	0.9941	0.9937	0.9933	0.9924
2012	0.9928	0.9930	0.9925	0.9928	0.9916	0.9895
2013	0.9942	0.9939	0.9934	0.9941	0.9928	0.9705
2014	0.9958	0.9955	0.9943	0.9954	0.9945	0.9048
